# Association Between Inter-Recti Distance and Impaired Abdominal Core Function in Post-Partum Women With Diastasis Recti Abdominis

**DOI:** 10.3389/jaws.2022.10909

**Published:** 2022-12-01

**Authors:** L. Bixo, G. Sandblom, J. Österberg, O. Stackelberg, K. Bewö, A. Olsson

**Affiliations:** ^1^ Department of Surgery, Mora Hospital, Mora, Sweden; ^2^ Department of Clinical Science and Education, Karolinska Institutet, Södersjukhuset, Stockholm, Sweden; ^3^ Department of Surgery, Södersjukhuset, Stockholm, Sweden; ^4^ Department of Clinical Sciences, Intervention and Technology (CLINTEC), Karolinska Institutet, Stockholm, Sweden; ^5^ Unit of Nutritional Epidemiology, Institute of Environmental Medicine, Karolinska Institutet, Stockholm, Sweden; ^6^ Stockholm Hernia Center, Stockholm, Sweden

**Keywords:** diastasis recti abdominis, rectus diastasis, post-partum, inter-recti distance, disability rating index

## Abstract

**Background and Aim:** The definition and management of Diastasis Recti Abdominis (DRA) is under debate. This study aimed to understand the correlation between the post-partum inter-recti distance (IRD) and functional impairments associated with core instability, with the hypothesis that IRD could serve as a proxy for core instability symptoms and constitute a tool in decision-making for DRA treatment.

**Material and Methods:** A cohort of post-partum women with abdominal core instability symptoms combined with DRA were studied. The size of IRD was measured with ultrasonography and cross-sectionally analysed against functional impairments registered with the self-report Disability Rating Index (DRI), which grades the ability to perform 12 different daily activities.

**Results:** A total of 224 women were included in the study. In univariable analysis, IRD was associated with impairment of the activities running (*p* = 0.007), heavy work (*p* = 0.036) and exercise/sports (*p* = 0.047), but not with dressing, walking, sitting for long periods, standing bent over a sink, carrying a suitcase, making a bed, light manual labour or heavy lifting. No significant correlations were seen in the multivariable analysis when adjustments were made for BMI and parity.

**Conclusion:** IRD and post-partum functional impairments had no significant correlation in multivariable analysis. The post-partum core instability condition is complex and probably associated with more factors than solely the IRD. The IRD alone does not seem to be a sufficient proxy for decision-making regarding optimal treatment. A more complete instrument to assess the post-partum abdominal core is warranted.

## Introduction

Diastasis recti abdominis (DRA) is an anatomical change of the abdominal wall with increased separation of the two rectus abdominis muscles (1). The condition often manifests as a vertical bulging of the anterior abdominal wall during muscle contraction (2). DRA typically represents a post-partum event among women (3). DRA in men is associated with increased intra-abdominal pressure such as visceral obesity (4). Owing to the mechanical stretching and hormonal changes that occur during pregnancy, increased interrecti distance is a normal and common physiological event among childbearing women (1), but may turn into a debilitating DRA condition. The DRA usually regresses after delivery but persists in 32%–46% (5,6,7). Recommended assessment methods for measuring the IRD are ultrasonography, callipers, or CT scan (8).

DRA is often associated with symptoms related to impaired abdominal core function such as trunk muscle weakness (9) and poor body image (10). It may also lead to lumbopelvic pain (1, 11) and urinary incontinence (12). The correlation between the persistent post-partum anatomical changes and the variety of functional symptoms is not fully understood and no generally accepted treatment is yet established (6, 13). Knowledge is insufficient regarding the consequences and management of DRA (7). The impairment is not considered a true hernia and there is consequently no risk for strangulation (14). However, it is associated with an increased risk of developing midline hernias (15,16,17).

The first-line treatment of DRA has traditionally consisted of non-invasive management (18, 19). Some studies show that physiotherapy is beneficial in patients with DRA but in general, the scientific quality is too poor to draw any conclusions (8, 20). If physiotherapy does not result in an adequate recovery, surgery is an alternative. Women who have undergone DRA surgery report an increased functional level and higher quality of life compared to before surgery (17,21,22,23,24,25,26). The purpose of surgical repair of the DRA is to restore the anatomy and thus re-establish abdominal core function. Improved abdominal wall contour is sometimes also lifted as a purpose, even in the absence of functional impairment. Several methods have been described with improved functional results (21, 27).

Although DRA has been a well-known phenomenon, it is only in recent years that surgical treatment with a focus on functional recovery has been considered. The potential functional benefits of surgical treatment and discussions of what previously has been considered as cosmetic surgery have led to significant media attention lately. Many women with DRA experience a lack of understanding from the public health care system regarding their symptoms and may turn to private clinics for surgical management (28).

There is a lack of knowledge regarding risk factors, clinical consequences, and management of DRA (29). Consequently, the accessibility and information to patients varies widely between different county councils and regions. A more standardised and evidence-based management regarding DRA is needed and suggested guidelines have been presented during the last years (8, 28, 30).

Functional impairment such as back pain, abdominal core instability, urinary incontinence and abdominal bulging is common following pregnancy. The ongoing debate is mainly focused on the DRA as a predictor for post-partum functional disabilities (18). Although women with functional impairment associated with DRA, have shown to benefit from surgery, the relationship between IRD and symptoms remains unclear. Some women with great IRD may lack symptoms of DRA. The present study, however, focused only on women with a history of symptoms that could be correlated to DRA.

This study aimed to investigate the association between the inter-recti distance (IRD) and self-reported functional disabilities measured with the Disability Rating Index questionnaire (DRI), with the hypothesis that inter-recti distance could serve as a proxy for abdominal core instability symptoms and constitute a tool in decision-making of DRA treatment.

## Methods

The study was based on a cohort of post-partum women with core instability symptoms combined with DRA. Participants were later considered either for core stability training or surgical repair of the DRA. They were recruited between January 2015 and June 2020. Demographic data, age, BMI, number of births and type of delivery [vaginal/caesarean section] was collected at baseline, as well as the IRD measured with ultrasonography. Some of the women in the present cohort were included in a previous study where they underwent surgery without any specific preoperative training program (17).

### Assessment of DRA

All participants were assessed with ultrasonography in a standardised procedure, in a supine position with knees flexed to 90° and relaxed abdominal muscles. The examination was performed with a 40 mm linear transducer. IRDs exceeding 40 mm were assessed with a panoramic view. The IRD was registered in mm at its widest point. An IRD exceeding 30 mm was considered as a potential surgical case (17). The width of 30 mm was chosen as it is widely accepted as a threshold for surgery. The examinations were performed by a senior surgeon with documented training in ultrasound examination, or by a radiologist. Results were recorded and saved in the patients’ medical records.

### Assessment of Functional Symptoms

**FIGURE 1:**
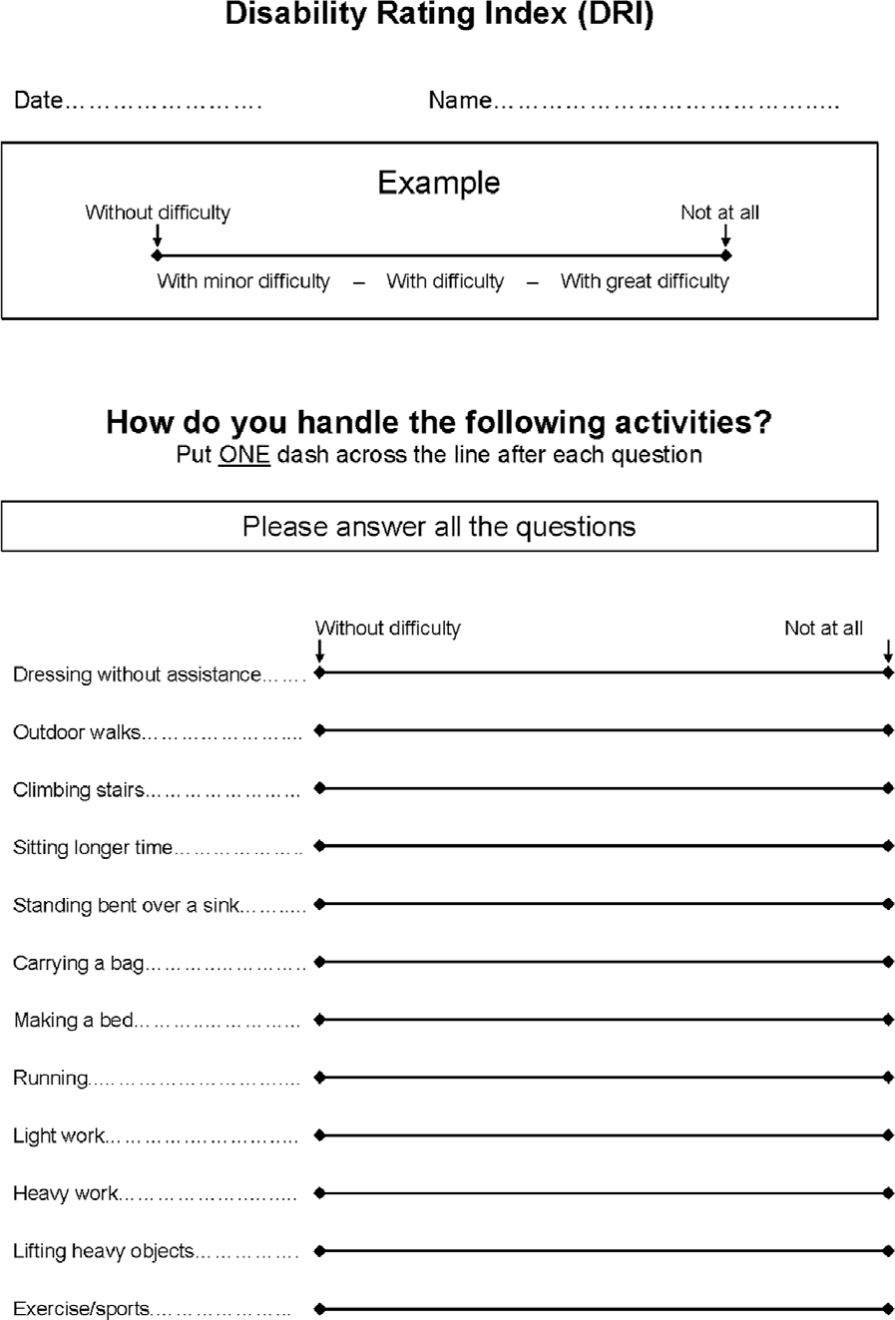
The Disability Rating Index (DRI) form covering twelve non-specific activities of daily life. The ability to perform each activity is registered by the participant on a visual analogue scale of 0–100 mm, where 0 represents no difficulty in performing the activity and 100 represents that the activity is impossible to perform.

Functional symptoms were registered with a self-report questionnaire, the Disability Rating Index (DRI), Figure 1 (31). The validated DRI covers twelve non-specific activities of daily life: dressing without assistance, outdoor walks, climbing stairs, sitting, standing bent over a sink, carrying a bag, making a bed, running, light work, heavy work, lifting heavy objects and exercise/sports. The ability to perform each activity is registered by the participant on a visual analogue scale of 0–100 mm, where 0 represents no difficulty in performing the activity and 100 represents that the activity is impossible to perform.

### Ethical Considerations

Written informed consent was obtained from all participants before inclusion. The Regional Ethics Committee, Karolinska Institutet, Stockholm, approved all procedures (Dnr. 2015/1753-31).

### Statistical Analysis

Continuous values for characteristics and DRI at the first visit are presented in medians with interquartile range (IQR). Differences of the categorised exposure (</≥3 cm IRD) were tested with the Kruskal-Wallis test. A linear regression model was used, with DRI as a dependent variable, to assess a possible association between DRI and IRD. Multivariable analyses were additionally adjusted for BMI and parity continuously. Two-sided *p*-values <0.05 were considered statistically significant. To test for non-linearity, DRI was modelled as a quadratic term.

## Results

A total of 224 women were included in this study. Of these, 208 women were examined with ultrasonography and completed the questionnaire at their first visit to the outpatient clinic, while sixteen completed the questionnaire retrospectively (two by letter and fourteen *via* telephone interview).

In this cohort, there was no difference in age, BMI, or parity when the IRD was categorised as </≥3 cm. Participants with an IRD ≥3 cm seemed to have more difficulties performing the specific tasks in the DRI compared with those with an IRD <3 cm (Table 1). No evidence of non-linearity was found.

**TABLE 1:** Characteristics, ultrasound values, DRI values of participants.

	Total (n = 224)	Interrecti distance		*p*-value^a^
		<3 cm (n = 8)	≥3 cm (n = 216)	
Characteristics				
Age, years	39 (21–67)	35.5 (34–44)	39 (21–67)	0.35
Body Mass Index, kg/m^2^	22.4 (17.0–36.0)	23.2 (19.5–29.6)	22.4 (17–36)	0.56
Parity	2 (1–5)	2 (2–2)	2 (1–5)	0.25
Vaginal	2 (0–5)	1 (0–2)	2 (0–5)	0.10
Sectio	1 (0–4)	1 (0–3)	1 (0–4)	0.94
Inter-recti distance, cm				
Ultrasound	4.5 (2.0–12.0)	2.3 (2–2.8)	4.8 (3–12)	(N/A)
Intraoperative, *n* = 164^b^	5 (3–13)	4.5	5 (3–13)	(N/A)
Abdominal Trunk Function Protocol				
Specific DRI (0-100p)				
Get dressed and undress without help	1 (0–81)	0 (0–7)	1 (0–81)	0.53
Taking walks	5 (0–95)	0 (0–45)	6 (0–95)	0.049
Walk on stairs	4 (0–94)	0 (0–45)	4 (0–94)	0.11
Sitting down for a longer period of time	24 (0–96)	0.5 (0–44)	25 (0–96)	0.011
Stand bent over doing dishes	38 (0–100)	1 (0–51)	40 (0–100)	0.005
Carry a suitcase or bag	24 (0–100)	1.5 (0–64)	27 (0–100)	0.066
Making the bed	11 (0–100)	0 (0–65)	13 (0–100)	0.051
Running	50 (0–100)	0 (0–77)	50 (0–100)	<0.001
Light manual labour	18 (0–100)	0 (0–51)	18 (0–100)	0.007
Heavy manual labour	53 (0–100)	3.5 (0–88)	55 (0–100)	<0.001
Heavy lifts	63 (0–100)	23.5 (0–100)	66 (0–100)	0.013
Exercise/sports	50 (0–100)	3.5 (0–78)	50 (0–100)	0.002
DRI points for the 7 lightest categories (0-700p)	121 (0–574)	15.5 (0–282)	130 (0–574)	0.019
DRI points for the 5 heaviest categories (0-500p)	234 (0–500)	44.5 (0–394)	243 (0–500)	0.003
Overall DRI points (0-1200p)	373 (0–1,063)	62.5 (0–676)	390 (0–1,063)	0.001

Values are medians (range) unless otherwise indicated.

^a^ *p*-values for differences across categories of IRD were obtained using the Kruskal-Wallis test.

^b^ N = 1 patient underwent surgery with an interrecti distance <3 cm.

**FIGURE 2:**
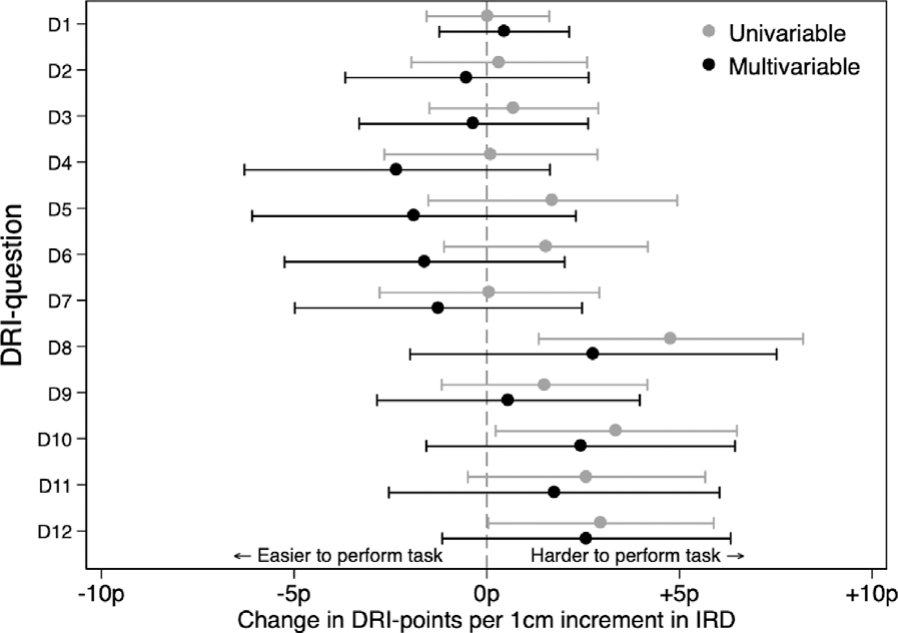
Linear regression analyses with DRI as dependent variable and DRI as independent. Multivariable were also adjusted for BMI and parity.

In univariable linear regression analysis, an increased DRI was associated with more difficulties in performing some of the heavier activities (e.g., running [*p* = 0.007], heavy work [*p* = 0.036] and exercise/sports [*p* = 0.047], visualized in Figure 2. However, the mean increase in DRI-score was small per centimetre increment in IRD (5p for running, 3p for heavy work, respectively 3p for exercise/sports, on a scale from 0 to 100). Combining the five heaviest categories, each one-cm increment in DRI was associated with a 14 p increase in DRI-score (*p* = 0.038). No association was observed between IRD and DRI when the linear model was adjusted for BMI and parity (*p* > 0.17, Table 2). Interpretation of the results was similar when restricting the analyses to participants with an IRD ≥5 cm and when categorising IRD </≥5 cm (data not shown).

**TABLE 2:** Association between IRD in cm and symptoms at first visit.

	Change in DRI-points for each cm increment in inter recti distance (95% CI)			
	Univariable	*p*	Multivariable^a^	*p*
Specific DRI^a^				
Get dressed and undress without help	0 (−2–2)	0.98	0 (−1–2)	0.59
Taking walks	1 (−1–3)	0.78	−1 (−4–3)	0.75
Walk on stairs	0 (−3–3)	0.53	−1 (−4–3)	0.75
Sitting down for a longer period of time	0 (−3–3)	0.94	−2 (−6–2)	0.25
Stand bent over doing dishes	2 (−2–5)	0.30	−2 (−6–2)	0.37
Carry a suitcase or bag	2 (−1–4)	0.25	−2 (−5–2)	0.38
Making the bed	0 (−3–3)	0.96	−1 (−5–2)	0.50
Running	5 (1–8)	0.007	3 (−2–8)	0.25
Light manual labour	1 (−1–4)	0.27	1 (−3–4)	0.74
Heavy manual labour	3 (0–6)	0.036	2 (−2–6)	0.23
Heavy lifts	3 (-0–6)	0.10	2 (−3–6)	0.42
Exercise/sports	3 (0–6)	0.047	3 (−1–6)	0.17
DRI points for the 7 lightest (Get dressed -> Making the bed)	4 (−10–18)	0.57	−7 (−25–11)	0.42
DRI points for the 5 heaviest (Running->Exercise/sports)	14 (1–28)	0.038	10 (−7–27)	0.25
Overall DRI points	19 (−5–44)	0.13	3 (−30–35)	0.87

^a^ Adjusted for Body Mass Index as a continuous variable, and number of parities.

## Discussion

Pregnancy often results in persistent anatomical changes of the abdominal wall, such as an increased inter-recti distance that can contribute to core instability, back pain and a poor body image.

DRA has been suggested to explain these functional impairments that affects a substantial proportion of the female post-partum population. The results of this study indicate a slight association between an increased IRD and impaired ability to perform more strenuous daily activities, but this effect fails to reach significance when covariates are considered. This study can therefore not present a significant correlation between DRA alone and self-reported functional impairments, indicating that the persistent post-partum core instability condition is more complex than solely caused by the widened linea alba. The findings of this study suggest that the DRA alone might not explain the panorama of physical symptoms associated with post-partum core instability.

The entire abdominal wall is progressively widened and stretched during pregnancy. A persistent deformation of the abdominal wall may affect the abdominal trunk function in various ways. A classification based on different myoaponeurotic deformities, including several anatomical changes, has been presented by Nahas (32) who concluded that abdominal wall protrusions are caused by the stretching of the entire abdominal wall and not only the linea alba. This is in line with our results; the pathogenesis of post-partum functional impairments is complex and cannot be explained by the DRA alone.

The different myofascial components of the abdominal trunk co-operate through fascial tension to maintain posture, stabilise the lumbar spine, enable motion and contribute to physiological functions (such as gastrointestinal and respiratory). Traditionally, a reduced IRD has been suggested to be the main focus of DRA rehabilitation (33), while studies focusing on general abdominal core function suggest that the transverse abdominal muscles (TrA) play an important role in maintaining abdominal wall tension (34, 35). Pre-activation of the TrA before a curl-up increases the abdominal wall tension and reduces the IRD-narrowing during the contraction, with less distortion of the linea alba. This may allow better force transfer between the flanks of the abdominal wall. The function of the abdominal muscles increases with TrA activation since it optimises tension of the linea alba despite reduced IRD narrowing (34, 35).

Multiple studies have shown that surgical repair of the DRA improves several functional disabilities (21). A surgical re-approximation of the widened linea alba reduces the abdominal circumference, which results in a stabilisation of the abdominal wall as well as a reduction of several functional symptoms (21). Although there is no significant correlation between IRD and DRI in this study there are several reports of functional improvements following surgical reduction of the inter-recti distance. Pieces of the puzzle are still lacking and there are obviously more aspects to this complex situation that need further investigation.

Post-partum core instability, causing back pain and abdominal muscle weakness, is well known by physiotherapists and personal trainers. There are numerous different training concepts recommended for reducing functional disabilities in the post-partum population (36). Multiple studies have reported both positive and negative results after non-specific training (20), while specific core stabilising training has been reported to provide an improved abdominal core function (18) and could be considered before surgical management of the DRA (8). The effect of core training is primarily to stabilise the abdominal canister and not reduce the inter-recti distance (28), although some studies have reported a decreased IRD following training (37).

The IRD is considered an important finding for deciding treatment but the definition of an abnormal IRD varies (8, 28, 30). Four of the 12 parameters tested with the DRI-scale (running, heavy work, lifting heavy objects and exercise/sports), showed a moderate correlation between the diastasis width and disability in performing the task in the univariable analysis, but there was no significant correlation between the width of the inter-recti distance and the degree of reported symptoms in the multivariable analysis. Our study suggests that even a modest IRD may have an impact on the associated functional impairments affecting daily life but the IRD alone does not seem to be sufficient to determine whether surgical reconstruction of a deformed abdominal wall is needed or not. However, although the current study only included eight participants with an IRD <3 cm, our data suggests that this group is less likely to be as functionally impaired as those with an IRD ≥3 cm.

Women with DRA perceiving functional impairment constitute a neglected patient group that deserve more attention. There is no consensus among health professionals on how to best approach the condition (38). Patient-reported outcome measures are valuable tools to evaluate clinical symptoms such as body image and core instability. When surgical repair is considered, a shared decision-making between the patient and the surgeon is crucial. A comparable situation is the management of osteoarthritis patients waiting for an arthroplasty. Osteoarthritis can be confirmed with radiology, but radiological findings generally do not correlate well with symptoms (39). Instead, the patient’s preferences play a significant role when deciding if a total hip or knee arthroplasty is necessary. Some patients wish to continue an active lifestyle whereas others request surgery to enable performance of simple daily living activities (40). This approach may apply also to diastasis recti abdominis.

## Limitations

This is a cross-section investigation without a control group. There were no data on concomitant midline hernias that might have had a confounding effect on reported functional disabilities. In twelve cases (5%), women who accepted inclusion fulfilled the DRI retrospectively. Therefore, we cannot exclude a response bias impact.

The participants’ activity levels have not been included in the calculations, possibly affecting the interpretation of the DRI reports. For someone who does not perform strenuous daily activities—the inability to perform these might not have been considered a problem when completing the DRI. Participants’ waistlines have not been recorded either. A certain IRD might constitute a larger problem for someone with a small waistline compared to someone with a greater waistline.

The cut-off level of 30 mm was chosen as it is widely accepted as a threshold for surgery. There is, however, no natural anatomical reason for choosing this level. When deciding on surgery, not only the width of the DRA should be taken into account.

As we did not have an *a priori* hypothesis regarding the relationship between the inter-recti distance and abdominal function, we did not do regular sample size estimation. It is possible that the present study is underpowered and that there may be a weak relationship that we were unable to detect. The uneven distribution, with only eight women with inter-recti distance less than 3 cm, also decreased the statistical power.

The outcome measures in the present study were purely subjective. Objective measures, e.g., trunk function assessed with Biodex^®^, may have provided more precise outcomes with higher inter-rater reliability.

As the aim of the study was to assess whether a predefined inter-recti distance could be set as a threshold for deciding on surgery, we analyse it as a dichotomous variable. However, linear analysis may have shown a significant correlation.

## Conclusion

Many post-partum women with persistent DRA suffer from functional disabilities. This study did not show any significant correlation between self-reported functional impairments and the inter-recti distance in isolation, even though there was a tendency towards an association between DRA and the ability to perform strenuous activities. The post-partum core instability condition is complex and consists of more components than solely the inter-recti distance. More research is needed to understand the interaction between anatomy and function, develop better assessment instruments and optimise the treatment. The inter-recti distance as well as other anatomical measures should also be evaluated in studies with greater statistical power than the present one.

## Data Availability

The raw data supporting the conclusion of this article will be made available by the authors, without undue reservation.
